# Engineering Brain Injury In Vitro: Human iPSC-Based Organoids in Microfluidic Systems

**DOI:** 10.3390/app16052309

**Published:** 2026-02-27

**Authors:** Satarupa Jena, Samuel Uzoechi, Cody Badeaux, Charity Johnson Campbell, Hailey Egido-Betancourt, Hala Madi, John Collins, Chioma Okey-Mbata, Qassim Dirar, Doo Yeon Kim, Sang Su Kwak, Sangho Ye, Salil Desai, Jin-Moo Lee, Daniel Laskowitz, Yeoheung Yun

**Affiliations:** 1 NERVE Center, FIT BEST Laboratory, Department of Chemical, Biological and Bio Engineering, North Carolina Agricultural and Technical State University, Greensboro, NC 27411, USA; 2 Biopico Systems Inc., 188 Technology Dr, Suite G, Irvine, CA 92618, USA; 3 Qorvo Inc., 7907 Piedmont Triad Pkwy, Greensboro, NC 27409, USA; 4 Genetics and Aging Research Unit, Mass General Hospital, Harvard Medical School, 114 16th Street, Charlestown, MA 02129, USA; 5 Department of Bioengineering, University of Pittsburgh, Pittsburgh, PA 15219, USA; 6 Industrial Engineering Department, North Carolina Agricultural and Technical State University, Greensboro, NC 27411, USA; 7 Department of Neurology, Washington University in St. Louis, St. Louis, MO 63110, USA; 8 Department of Neurology, Duke University Medical Center, Durham, NC 27710, USA

**Keywords:** traumatic brain injury, microfluidics, human induced pluripotent stem cells, neurovascular units, drug discovery

## Abstract

Traumatic brain injury (TBI) presents a major biomedical challenge due to its complex biomechanics and the heterogeneous cellular responses it elicits, including neuronal death, glial activation, and blood–brain barrier disruption. Traditional in vitro models, including 2D neuronal cultures, brain slices and transwell systems, have provided valuable insights into molecular and cellular biology but remain limited by their lack of human-specific architecture, vascularization, and neurovascular interactions. The purpose of this review is to systematically examine advances in in vitro TBI modeling, with particular attention to studies leveraging human induced pluripotent stem cell (iPSC)-derived neural and vascular tissues, organoids, hydrogel scaffolds, microfluidic platforms, and mechanical injury. We highlight how the integration of neurovascular unit (NVU) components has improved the physiological and functional relevance of these models. Finally, we identify key limitations, including variability in organoid maturation, incomplete vascularization, and lack of methodological standardization, and outline future directions for improving translational fidelity. Therefore, this review contributes to a critical evaluation of emerging technologies and their potential to advance neurotrauma research and therapeutic discovery.

## 1. Introduction

Traumatic brain injury (TBI) is a major cause of death and long-term disability, with an estimated 20.8 million new cases annually and accounting for over 5 million years lived with disability (YLDs) worldwide in 2021 [1,2]. The global burden continues to rise due to increased motorization, sports participation, and military conflicts. Pathophysiology is complex, involving cascades of mechanical stress, excitotoxicity, oxidative stress, neuroinflammation, blood–brain barrier (BBB) disruption, and progressive neurodegeneration [3,4]. In vivo models of TBI, such as fluid percussion injury (FPI), weight drop/impact acceleration, blast-induced TBI, and controlled cortical impact (CCI), replicate focal injuries, while diffuse injuries are modeled by shear deformation or rapid acceleration leading to traumatic axonal injury and edema [5]. Classic two-dimensional (2D) in vitro models provided insight into excitotoxicity and apoptosis but cannot capture the three-dimensional cytoarchitecture and cellular heterogeneity of the human brain [6]. Although informative, these systems face limitations including interspecific differences, variability, and reliance on terminal endpoints.

**Figure 1.**
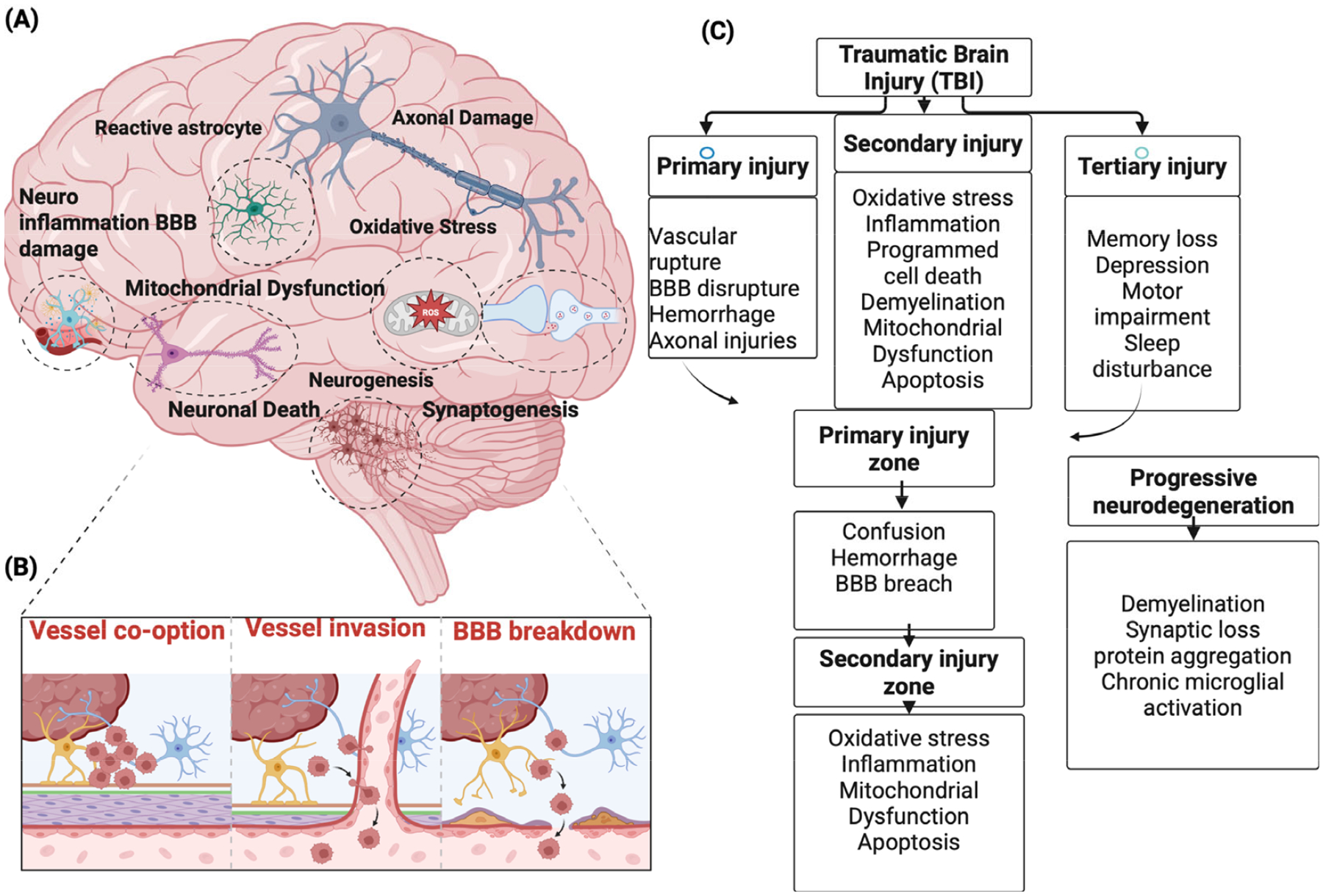
Comprehensive Schematic of TBI-Induced Cellular, Vascular, and Degenerative Processes: (**A**) shows the cellular and molecular consequences of traumatic brain injury, including reactive astrocytes, axonal damage, neuroinflammation with BBB disruption, oxidative stress, mitochondrial dysfunction, neuronal death, and limited repair responses such as neurogenesis and synaptogenesis. (**B**) depicts the progression of vascular pathology from vessel co-option to vessel invasion and ultimately BBB breakdown, highlighting how vascular instability drives inflammation and secondary damage. (**C**) summarizes the temporal injury cascade, moving from primary mechanical trauma to secondary biochemical responses and finally tertiary neurodegenerative outcomes, including chronic demyelination, synaptic loss, and microglial activation. Figure created with BioRender (www.biorender.com).

There is a growing societal need for more ethical, effective, and human-relevant biomedical research of national priority. Public concern over animal testing and its limitations is driving demand for alternatives. Emerging technologies—such as CRISPR-Cas-9, iPSCs, Organoids, Tissue Chips, and Artificial Intelligence (AI) models—offer more accurate and personalized insights into human health. Recently, NIH intended to establish the Office of Research Innovation, Validation, and Application (ORIVA), which coordinates NIH-wide efforts to develop, validate, and scale the use of non-animal approaches across the agency’s biomedical research portfolio and serves as a hub for interagency coordination and regulatory translation for public health protection [7,8]. Similarly, the Food and Drug Administration (FDA) takes a groundbreaking role in the development of drugs with more effective, human-relevant methods [9]. This review evaluates recent progress in vitro mechanical injury models, human iPSC-derived spheroids/organoids, hydrogel scaffolds, and functional readouts such as microelectrode arrays (MEAs) [10–12]. We highlight current methodologies, key challenges including reproducibility and vascular perfusion, and future opportunities for developing next-generation in vitro TBI platforms [13–16]. Within this broader methodological landscape, clarifying the translational scope of iPSC-derived in vitro systems is essential. Within this broader methodological landscape, clarifying the translational scope of iPSC-derived in vitro systems is essential. Instead, they preferentially model cellular- and tissue-scale injury processes, including diffuse axonal injury, repetitive sub-concussive loading, microvascular dysfunction, and secondary injury cascades, under highly controlled conditions. 2. TBI pathophysiology: TBI can be characterized by three phases (Figure 1); (1) Primary injury occurs at the moment of impact and reflects the mechanical forces applied to the head, producing cortical contusions, skull fracture, intracranial hemorrhage, and especially diffuse axonal injury (DAI), in which shear and tensile forces disrupt axons at the gray, white matter junction, corpus callosum, and brainstem. This stage often disrupts the blood–brain barrier (BBB) and cerebral blood flow, producing immediate ischemia and tissue deformation. (2) Secondary injury evolves over minutes to days following the initial insult and involves a complex cascade of pathophysiological processes, including cerebral blood flow disturbances, ionic imbalance, glutamate-mediated excitotoxicity, intracellular Ca^2+^ overload, mitochondrial dysfunction, oxidative and nitrative stress, BBB breakdown, and activation of microglia and astrocytes. Activated microglia and astrocytes release proinflammatory cytokines and chemokines, while infiltrating immune cells further compromise the BBB and promote vasogenic and cytotoxic edema, leading to increased intracranial pressure and reduced perfusion. These secondary insults, such as hypoxia, hypotension, hypercapnia, hyperglycemia, pyrexia, and raised intracranial pressure, further amplify this cascade and are strongly associated with worse functional outcomes in severe TBI. (3) Tertiary injury develops over the long term and is characterized by chronic pathological changes such as gliosis, Wallerian degeneration, demyelination, and persistent white matter loss [17]. Chronic inflammation and vascular dysregulation contribute to sustained BBB leakage and microbleeds, while abnormal protein aggregation (e.g., hyperphosphorylated tau and amyloid-β) links TBI to long-term neurodegenerative disorders such as chronic traumatic encephalopathy (CTE) and Alzheimer’s disease (AD). A clear understanding of how to reproduce strategies of TBI and the corresponding pathophysiological mechanisms is essential for developing accurate and translational models.

## 2. Human iPSC-Derived Organoids and Neurovascular Unit for TBI Modeling

**Figure 2.**
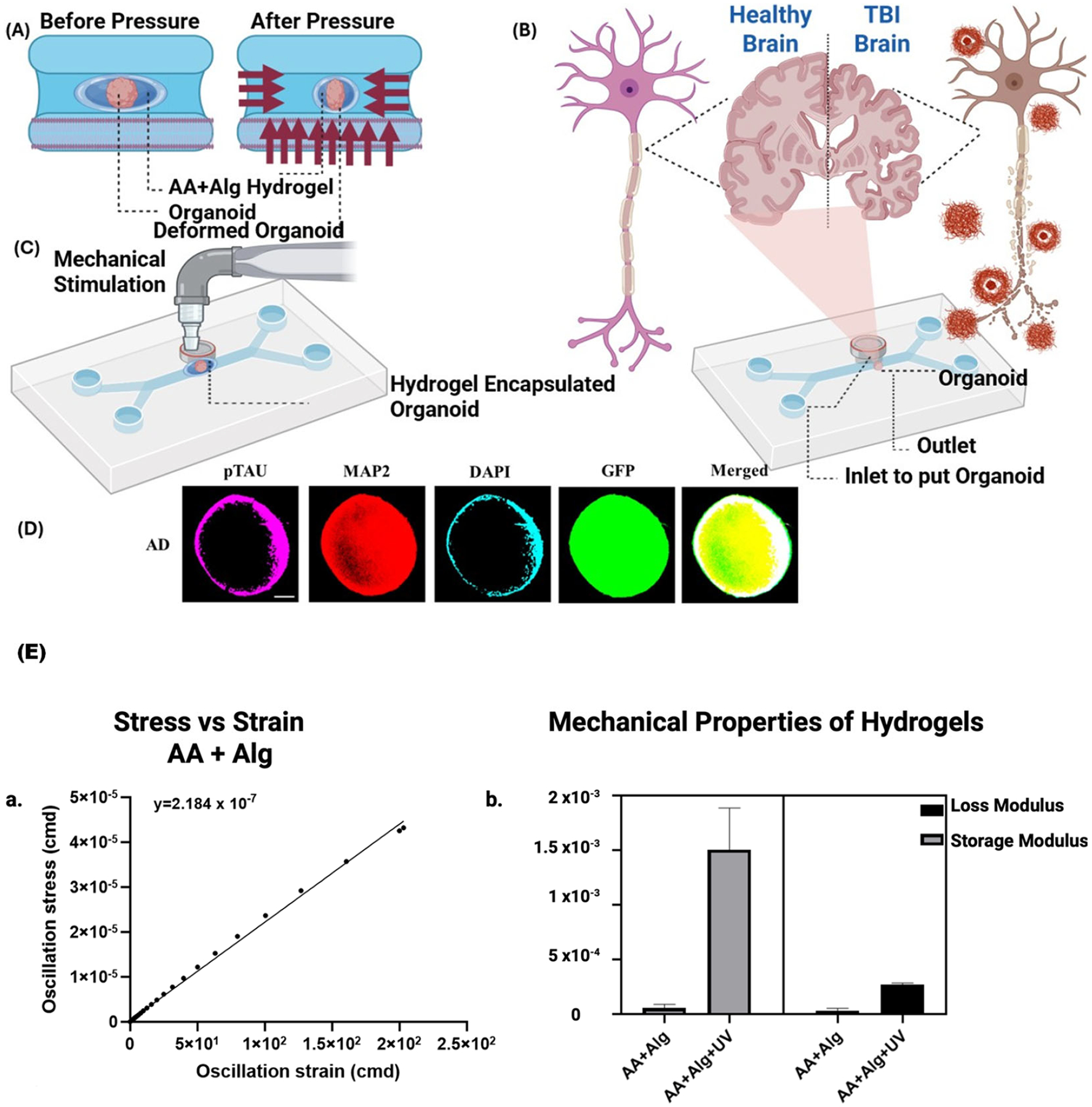
Controlled Mechanical Injury Using PDMS-Based Microfluidic Platforms: (**A**) flexible PDMS membranes integrated with pressure controllers then deliver precise mechanical stimuli allowing reproducible injury application, (**B**,**C**) cross-sectional view highlighting the deformation of PDMS membranes upon pressure actuation, simulating compressive shear forces relevant to traumatic brain injury and before and after pressure mechanical stimuli allowing reproducible injury application, and (**D**) represented stained image of AD spheroid under compression (p-Tau, MAP2, and DAPI), (**E**) Mechanical testing of hybrid hydrogels under varied conditions, including stress–stretch behavior (**a**), which shows a linear stress–strain relationship for the AA + Alg formulation with a calculated slope of 2.184 × 10^−7^, indicating a low elastic modulus and a soft matrix suitable for neural tissue engineering. The right panel (**b**) compares the storage and loss moduli of AA + Alg and UV-crosslinked AA + Alg hydrogels, demonstrating that UV treatment substantially increases stiffness through an elevated storage modulus while also enhancing viscous behavior via an increased loss modulus, thereby improving mechanical integrity for microfluidic applications. Together, these results highlight tunable mechanical properties achieved through UV crosslinking, enabling the design of brain-like matrices for neurospheroid encapsulation.

Human iPSC-derived neural systems and brain organoids have emerged as powerful platforms for modeling traumatic brain injury (TBI) because they provide human-specific cellular architecture, genetic backgrounds, and mechanistic responses that are not captured in traditional animal models [18–21]. These systems enable interrogation of injury responses within genetically defined human neural tissues under controlled experimental conditions. Figure 2 provides an overview of this integrated platform, illustrating the reprogramming of somatic cells into iPSCs, their differentiation into neural and vascular lineages, and assembly into a microfluidic NVU-on-chip containing neurons, astrocytes, microglia, endothelial cells, pericytes, and immune cells relevant to TBI pathology.

Early iPSC-based 2D models showed that mechanically stretching hiPSC-derived neurons reproduces hallmark TBI phenotypes such as neurite degeneration, cytoskeletal disruption, calcium dysregulation, and cell death [22], recapitulating primary injury responses. More recently, three-dimensional (3D) iPSC-derived cerebral organoids have been used to model TBI-relevant injury mechanisms in vitro. Applying controlled cortical impact (CCI) or mechanical compression to organoids induces neuronal loss, astroglia activation, metabolic dysfunction, and injury-linked transcriptional changes, closely matching human TBI pathology [23]. For example, blast-wave exposure and mechanical insult in cortical organoids have been used to model mild and repeated TBI, revealing long-term structural and molecular alterations, as well as TDP-43-related dysfunction relevant to chronic neurodegeneration [24].

Despite these advantages, iPSC-derived neurospheroids and cerebral organoids remain developmentally immature, most closely resembling fetal or early postnatal stages of the human brain. Consequently, they do not fully recapitulate adult brain features such as extensive myelination, mature synaptic pruning, region-specific vascular specialization, or long-range connectivity. This developmental state can influence cellular injury responses, inflammatory signaling, and functional recovery following mechanical insults. As a result, current organoid-based TBI models are particularly well suited for investigating early mechanobiological responses, neurodevelopmental vulnerability, and secondary injury processes rather than fully modeling adult-onset or chronic TBI pathology.

Human iPSC and organoid platforms uniquely capture human-relevant mechanisms of both primary mechanical injury and evolving secondary pathology, offering scalable, genetically defined, and ethically accessible models for understanding TBI and testing therapeutics. Endothelial differentiation revealed that VEGF- and FGF2-driven lineages form lumenized, perfusable vasculature expressing CD31, VE-cadherin, and vWF. These findings were further expanded by studies demonstrating that iPSC-derived pericytes support barrier integrity and vascular stability [25]. In parallel, the generation of iPSC-derived microglia exhibits immune surveillance and inflammatory responses [26]. Together, these advances demonstrate that iPSC-derived organoids can reproduce key neural, vascular, and immune components of the neurovascular unit (NVU), providing a human-relevant system to interrogate TBI pathology and evaluate therapeutic strategies [27–29].

NVU-integrated and vascularized organoids can capture the complex interplay between neurons, glial cells, and the cerebral vasculature by integrating endothelial cells, astrocytes, and pericytes to mimic the blood–brain barrier (BBB) structure functions. Importantly, these platforms enable direct investigation of BBB disruption, neurovascular uncoupling, and inflammatory signaling in response to mechanical injury [30]. Vascularized assembloids, developed by fusing brain and vascular organoids, were shown to support BBB-like function, neuroinflammation, and cellular injury mechanisms in human-relevant systems [15,31].

At the same time, the self-organizing features that confer biological complexity and human relevance also introduce experimental variability. Organoid-based systems inherently exhibit structural heterogeneity and limited reproducibility. Because self-organization governs tissue assembly, substantial variability in size, morphology, and cellular composition can occur between batches and even within the same experiment. Such heterogeneity may influence mechanical strain distribution, injury thresholds, and downstream molecular or functional readouts, complicating quantitative comparison across studies. Explicit recognition of these factors is therefore essential when interpreting injury outcomes and assessing the robustness and translational relevance of in vitro TBI models.

## 3. Microfluidic TBI Modeling

Microfluidics/tissue chips enable TBI modeling by enabling precise mechanical control over neural cells, both at the single-cell level and in the three-dimensional (3D) tissues comprising multiple cell types, such as neurospheroids/organoids derived from induced pluripotent stem cells (iPSCs) (Table 1). The microfluidics offers precise control using microchannels and malleable membranes to impart controlled strain, compression, or shear forces, mimicking injury scenarios ranging from acute focal impacts to sustained compressive loads while maintaining cell viability through perfusion systems [32]. For example, microfluidic devices allow (1) axonal stretch/diffuse axonal injury on chips [33], (2) microfluidic NVU chips for TBI [34], and (3) blast-like or shear injury microfluidics [35]. Microfluidics is leveraged to apply strain specifically to regional neuronal compartments, such as axons, enabling a detailed examination of diffuse axonal injury mechanisms. Microfluidic chip initially used for diffuse axonal injury (DAI), the hallmark of mild to moderate TBI, demonstrating neurite beading, cytoskeletal breakdown, and degeneration, while thicker axons resisted deformation [36]. Pan and colleagues were motivated by the clinical gap in modeling non-disruptive, sub-concussive axonal injuries that accumulate over time. The “axons-on-a-chip” applied microfluidic shear jets to axons, producing focal swellings, calcium influx, and periodic cytoskeleton disruptions without causing outright cell death [37]. This revealed that early TBI involves calcium-dependent mechanotransduction pathways leading to cytoskeletal disorganization, providing a mechanistic basis for why repeated sub-concussive hits can impair neuronal integrity. Because TBI patients often present with systemic complications, several groups moved toward multiorgan-on-chip approaches. The brain–lung axis was reported to show that brain injury can trigger neurogenic pulmonary edema and immune cell trafficking, while lung inflammation can worsen cerebral pathology [38,39]. These multi-organ platforms are used to trace cytokine storms, immune activation, and vascular permeability changes between the brain and lung compartments. Similarly, human neurospheres with liver equivalents showed that neural and hepatic tissues are maintained in perfused co-culture for two weeks [40]. This opened the door to exploring how liver metabolism, detoxification, and acute-phase protein secretion might influence recovery and secondary injury after TBI.

Within this framework, iPSC-derived neurospheroid- and organoid-based microfluidic platforms enable controlled interrogation of injury mechanisms characteristic of mild-to-moderate traumatic brain injury, including diffuse axonal injury, repetitive subconcussive loading, and microvascular dysfunction. Microfluidic stretch, compression, and shear systems isolate specific biomechanical components of rapid acceleration–deceleration and blast exposure, allowing direct linkage between applied mechanical stimuli and cellular or tissue-level injury responses that are difficult to decouple in vivo. Moreover, microfluidic confinement and hydrogel encapsulation partially mitigate reproducibility challenges inherent to organoid systems by standardizing tissue geometry, mechanical loading profiles, and biochemical gradients. Quantitative functional endpoints, particularly MEA-based electrophysiology, provide longitudinal readouts that are less sensitive to morphological variability alone, thereby improving experimental robustness and cross-study comparability. Nevertheless, variability in neurospheroid or organoid size remains an important contributor to heterogeneity in local strain and stress distributions, and hydrogel encapsulation alone does not fully eliminate size-dependent differences in mechanical readouts. Accordingly, careful control of initial spheroid dimensions, normalization strategies, and reporting of size distributions remain essential for accurate interpretation of mechanically induced injury responses.

## 4. Extracellular Matrix in TBI Modeling

Hydrogels play a central role in microfluidic and tissue-engineered TBI models, providing (1) structural/mechanical mimicry of the brain, (2) biochemical microenvironment for neural and glial cells, (3) controlled perfusion, and (4) tunable biophysical and chemical properties [48]. The brain has an elastic modulus in the range of 0.1–1 kPa. Hydrogels such as gelatin methacrylate (GelMA), Matrigel, collagen, fibrin, alginate, and hyaluronic acid (HA) can be tuned to match this stiffness, allowing them to serve as structurally and mechanically relevant scaffolds for organoids/spheroids culture, such as guiding radial organization, neural rosette formation, and maintaining polarity through controlled growth factor gradients [49]. Matrigel-encapsulated organoids were matured to ventricular-like zones and layered neuroepithelium, forming rudimentary circuits with spontaneous electrical activity [50]. Incorporation of vascular and immune elements, including endothelial cells, pericytes, and iPSC-derived microglia, enables synaptic remodeling, cytokine secretion, and immune surveillance, enhancing physiological relevance [51]. Hydrogel scaffolds maintain 3D tissue architecture and provide biomimetic mechanical and biochemical cues, supporting cortical organoids, multi-region assembloids, and neurospheroids [52,53]. Hydrogels transmit controlled shear, stretch, or compression forces in microfluidic platforms, enabling precise modeling of traumatic brain injury (TBI) [54]. Natural hydrogels, such as collagen, Matrigel, and hyaluronic acid, provide bioactivity but are variable, whereas synthetic hydrogels like polyethylene glycol (PEG) offer tunable stiffness and reproducibility. Hybrid hydrogels, notably alginate–acrylamide (AA + Alg), combine biocompatibility and mechanical robustness, facilitating the study of mechanobiological responses and neurodegenerative processes [55,56].

Figure 2 highlights how the Acrylamide (AA) and Alginate (Alg) hydrogel system behaves mechanically, showing its potential to realistically mimic brain tissue. The stress and strain curve reveals a clear linear pattern, meaning the hydrogel stretches predictably without breaking, an important quality when studying how brain cells respond to physical forces. The graph compares the hydrogel’s behavior before and after UV crosslinking. After crosslinking, the hybrid Acrylamide (AA) and Alginate (Alg) hydrogel becomes stiffer and more elastic, which shows the stronger bonds forming between the polymers. To model traumatic brain injury (TBI), neurospheroids were encapsulated with the Acrylamide (AA) and Alginate (Alg) hydrogel and gently inserted into microfluidic channels [57,58]. The immunocytochemistry images showcase the key pathological hallmarks of traumatic brain injury (TBI), including phosphorylated tau (pTau) accumulation, disrupted neural connections, and changes in glial and vascular activity [19,59–61].

**Figure 3.**
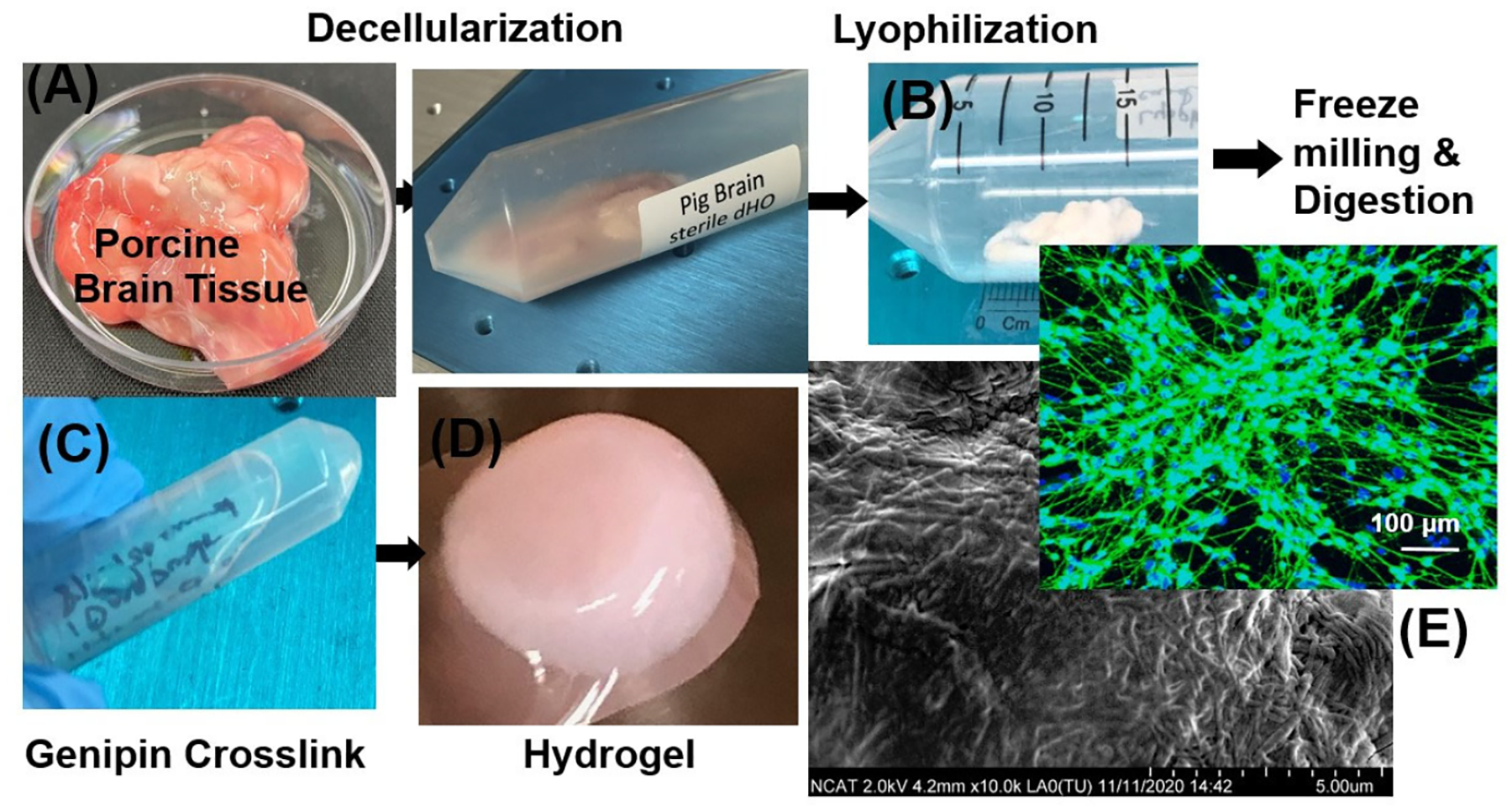
Development and characterization of porcine brain–derived extracellular matrix (ECM): (**A**) Fresh porcine brain tissue following decellularization to remove cellular and immunogenic components while preserving native ECM architecture. (**B**) Decellularized tissue is lyophilized, mechanically milled into a fine powder, and enzymatically digested to generate a solubilized ECM suspension. (**C**) ECM solution is crosslinked with genipin to enhance mechanical stability and structural integrity. (**D**) Crosslinked material undergoes thermal gelation to form a self-supporting hydrogel suitable for neural tissue engineering applications. (**E**) Representative fluorescence micrograph showing neuronal cells interacting with the ECM hydrogel (scale bar, 100 μm), alongside scanning electron microscopy revealing the porous microstructure that supports cell attachment and neurite extension (scale bar, 5 μm).

This hydrogel-based microfluidic setup allows for real-time monitoring, reproducible injury delivery, and controlled biochemical gradients, enhancing the physiological relevance of iPSC-derived neurospheroids. Together, this approach provides a powerful platform for studying TBI pathophysiology and screening potential therapeutics with high precision and reproducibility [62]. Common approaches include thermal gelation, ionic crosslinking, and photo-crosslinking, each offering distinct control over scaffold mechanics and temporal stability [63,64]. Such tunability is essential to approximate the biomechanical environment of human brain tissue, which exhibits an elastic modulus of ~1 kPa and a storage modulus (G′) between 140 and 620 Pa, varying with age and region [48] (Table 2). Our group also generates hydrogel using porcine brain–derived ECM, which involves (A) decellularizing porcine brain tissue, (B) lyophilizing, freeze-milling, and digesting the ECM, (C) crosslinking with genipin, (D) forming the ECM hydrogel, and (E) integrating neuronal tissue within the resulting matrix (Figure 3) [65].

Table 2 highlights how hydrogel stiffness correlates with lineage-specific outcomes. Neural stem cells favor a softer microenvironment (~0.1–1 kPa) that promotes neuronal differentiation, while stiffer substrates (>1 kPa) bias cells toward glial fates [41]. Matrigel, a thermo-gelled ECM, provides an elastic modulus of ~0.5–2 kPa, supporting neural rosette formation and cortical organization. GelMA, with a tunable modulus from 0.5 to 20 kPa via photo-crosslinking, enables patterned stiffness gradients for advanced tissue engineering [42,62]. Alginate scaffolds, crosslinked ionically, span 0.1–10 kPa and allow reversible modulation suitable for dynamic mechanical studies. PEG-based hydrogels, although biologically inert, provide highly tunable stiffness and chemical customization, making them versatile for hybrid designs [66]. Relating scaffold mechanics to pathology, brain deformation in mild-to-moderate TBI corresponds to approximately 5–20% strain, with peak stresses transmitted across neural and vascular interfaces [67]. Hydrogels engineered within the physiologic viscoelastic range (G′ ~140–620 Pa) provide a controllable platform for reproducing these conditions. By selecting appropriate crosslinking strategies, researchers can design scaffolds that maintain long-term culture stability, support organoid and neurospheroid maturation, and mechanistically model excitotoxic, vascular, and inflammatory cascades relevant to TBI and neurodegeneration [63].

## 5. Tissue Clearing, Expansion, and Visualization

Advancing organoid and microfluidic models of traumatic brain injury (TBI) require visualization strategies that resolve both large-scale tissue organization and nanoscale features. Tissue clearing methods such as CLARITY, PACT, CUBIC, and SHIELD reduce light scattering while preserving fluorescent signals, enabling volumetric immunolabeling of neurons, glia, and vascular networks within intact organoids [40,44]. In a typical workflow, the sample is fixed and labeled, permeated with monomers and an anchoring reagent, then polymerized into a dense hydrogel. Unanchored proteins are partially digested, and the gel–tissue composite is immersed in water, swelling uniformly, usually about fourfold in each dimension, while preserving relative spatial organization. The expanded, optically clear sample can then be imaged by confocal or light-sheet microscopy at effective resolutions of 60–80 nm [43,44,49]. Expansion microscopy (ExM) further extends resolution by physically enlarging tissues, facilitating nanoscale imaging of synapses, axonal varicosities, and glial interactions with conventional microscopes [45–47,68]. Light-sheet fluorescence microscopy (LSFM) complements these approaches by enabling rapid, gentle, and high-resolution visualization of cleared or expanded tissues [49]. Applied to TBI models, LSFM reveals axonal degeneration, glial activation, vascular remodeling, and neuroinflammation that evolve across acute and chronic phases, closely paralleling clinical observations [50–52]. Clearing and expansion are particularly powerful when correlated with functional recordings from microelectrode arrays (MEAs). MEAs quantify electrophysiological responses to injury, including changes in firing rate, burst activity, and synchrony [53,55]. However, electrical readouts cannot resolve the structural underpinnings of these changes. By applying clearing or ExM to MEA-coupled constructs, researchers can directly map electrophysiological deficits, such as reduced synchrony or impaired network oscillations, to underlying synapse loss, dendritic retraction, or glial infiltration [47,56,68]. This multimodal integration bridges the gap between functional decline and structural pathology, providing a more complete picture of TBI mechanisms. Within microfluidic TBI platforms, hydrogel encapsulation stabilizes organoids during controlled perfusion or pressure-induced injuries. Adapted clearing protocols ensure that both neural and vascular compartments remain optically accessible, enabling structural-functional correlation across injured 3D systems [69,70]. Together, clearing, expansion, and MEA integration create a multiscale pipeline for investigating neurodegeneration, vascular remodeling, and immune interactions in human-relevant models of TBI, enhancing mechanistic understanding and supporting therapeutic discovery [43,44,49,71].

As summarized in Table 3, in vitro and microphysiological platforms now span a broad continuum of complexity, enabling researchers to model distinct biomechanical and cellular aspects of traumatic brain injury (TBI) with increasing fidelity. Reductionist systems such as hiPSC-derived neuronal or glial cultures provide high-resolution access to acute cellular responses but lack the three-dimensional cytoarchitecture and vascular cues that influence injury propagation. Microfluidic stretch and compression platforms introduce controlled mechanical loading, allowing precise interrogation of axonal strain, shear forces, and focal deformation. More advanced brain-on-chip and axon-on-chip systems incorporate compartmentalized circuits, fluid flow, and barrier interfaces, thereby improving physiological relevance while maintaining compatibility with imaging, electrophysiology, and high-content assays. Human cortical organoids, vascularized assembloids, and mechanically loaded or blast-exposed organoid models further recapitulate layered architecture, long-range connectivity, and chronic neuroinflammatory dynamics characteristic of TBI. Multi-organ chips, including brain–lung and brain–liver platforms, extend this capability by enabling the study of systemic interactions and secondary injury cascades. Collectively, the models outlined in Table 3 provide a scalable experimental landscape from simple to highly biomimetic, supporting mechanistic discovery, therapeutic screening, and translational evaluation of TBI pathophysiology.

## 6. Functional Readouts Using Microelectrode Arrays (MEAs)

**Figure 4.**
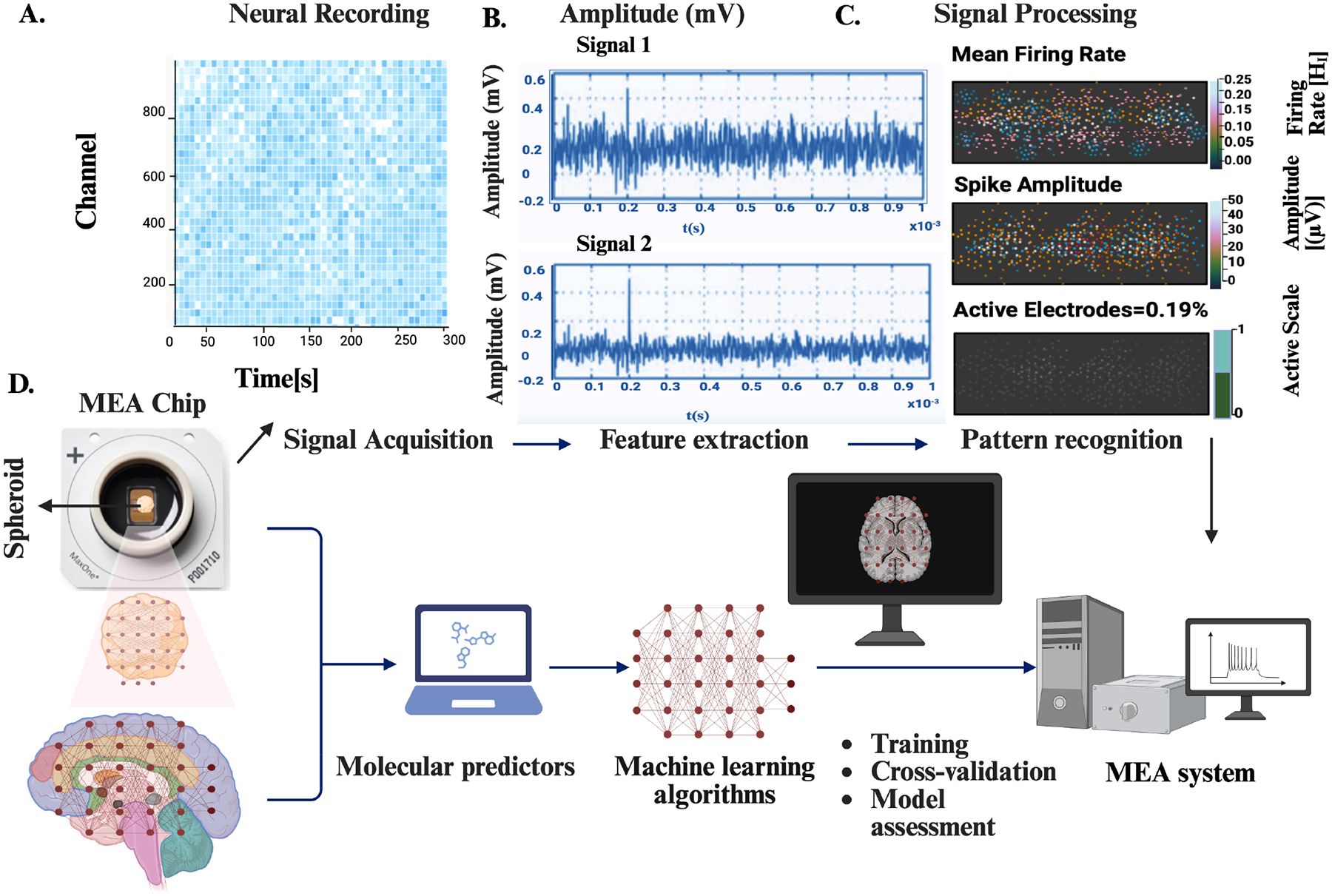
MEA-Based Neural Activity Acquisition, Feature Extraction, and Computational Analysis Workflow: This figure illustrates the complete workflow of MEA-based neural activity acquisition and computational analysis. (**A**) shows a neural activity heatmap, where each row represents an electrode channel, and color intensity reflects spontaneous firing patterns across the network. (**B**) presents representative extracellular voltage traces from two electrodes, demonstrating typical spike amplitudes and temporal firing dynamics used for downstream analysis. (**C**) summarizes key extracted electrophysiological features, including mean firing rate, spike amplitude distribution, and the proportion of active electrodes, providing a quantitative overview of network excitability and culture viability. (**D**) outlines the integrated MEA machine learning pipeline, beginning with spheroid placement on the MEA chip, followed by signal acquisition, preprocessing, feature extraction, and pattern recognition, culminating in predictive modeling and functional assessment through machine-learning algorithms. Figure created with BioRender (www.biorender.com).

Functional assessment remains a critical benchmark in the development of in vitro traumatic brain injury (TBI) models (Figure 4). While imaging, biochemical assays, and immunohistochemistry provide valuable insight into molecular pathology, these approaches are largely static and endpoint in nature. Neural function, however, is inherently dynamic, characterized by coordinated electrical activity across complex networks. Capturing these electrophysiological signatures is essential to bridge cellular injury responses with system-level dysfunction. Microelectrode arrays (MEAs) have therefore become indispensable tools, offering non-invasive, real-time, and longitudinal monitoring of neuronal network activity that complements structural and molecular readouts [53,72,73].

MEAs consist of microfabricated electrode grids capable of recording extracellular field potentials from multiple sites simultaneously [74]. Unlike single-cell patch-clamp techniques, MEAs enable parallel sampling of population-level activity over extended timeframes, capturing changes in spiking, bursting, and synchrony [75,76]. These metrics directly reflect alterations in network connectivity and excitability, which are frequently disrupted following TBI. For example, studies have reported decreased firing rates, impaired burst organization, and delayed recovery of coordinated activity in hydrogel-encapsulated neurospheroids exposed to controlled mechanical deformation, phenomena consistent with electrophysiological disruptions observed in injured brains in vivo [77]. Integration of MEAs with hydrogel and organoid-based systems provides a physiologically relevant platform to interrogate injury outcomes. Hydrogels such as collagen, alginate, and PEG derivatives maintain 3D cytoarchitecture and mechanical stability, while neurospheroids and organoids offer human-derived cellular complexity [56,78]. Yet the fidelity of these constructs cannot be judged by morphology alone; functional integration is equally essential. MEAs allow researchers to determine whether organoid-derived neurons form coherent networks capable of sustaining activity, and how these networks respond to traumatic insults. This is particularly powerful when comparing hydrogel formulations, where subtle differences in stiffness or biochemical composition can alter excitability and resilience [79].

Recent advances have further expanded the reach of MEA technology. Three-dimensional electrode arrays, penetrating microelectrodes, and flexible or stretchable MEAs now interface more effectively with volumetric tissues, enabling recordings from both surface and deeper neuronal layers [80–82]. These devices are increasingly integrated with microfluidic injury platforms, where hydrogel-encapsulated spheroids are positioned adjacent to pressure chambers and subjected to controlled deformation [83,84]. Such setups mimic clinically relevant injury regimes, ranging from single high-magnitude pulses that simulate traffic accidents or blast injuries to repeated moderate insults that model concussions common in athletics or military training [69,70,85]. Critically, MEAs can record network responses during and after these mechanical perturbations, capturing dynamic injury signatures in real time [86]. The scalability of MEAs also aligns with translational needs. Commercial multiwell MEA plates enable high-throughput screening of injury parameters, hydrogel conditions, and pharmacological interventions [73,87]. For instance, neuroprotective compounds can be evaluated by monitoring their ability to restore firing rates or synchrony in injured networks. Coupling electrophysiological data with biomarker sampling, calcium imaging, and immunostaining provides multimodal insights into how trauma reshapes neural circuits, glial responses, and inflammatory cascades [88,89].

Despite challenges, including signal variability due to electrode–cell proximity and limited spatial resolution, MEAs remain the most accessible platform for functional readouts in 3D TBI models [90]. Innovations such as transparent and optically compatible MEAs, or microfluidic-integrated stretchable electrodes, are bringing the technology closer to in vivo relevance. By anchoring molecular and structural findings within the framework of network performance, MEAs provide translationally meaningful outcomes that parallel patient deficits. As organoid and microfluidic injury models advance, MEAs will continue to serve as essential platforms for probing electrophysiological dysfunction and testing therapeutic interventions aimed at mitigating the devastating consequences of brain injury [91–93].

## 7. Conclusions and Future Directions

Microengineered systems have transformed the study of traumatic brain injury (TBI), enabling controlled replication of mechanical stimuli and cellular responses that were previously inaccessible in conventional models. As outlined in Table 2, these platforms, ranging from single-organ microfluidic chips to multi-organ and organoid-on-chip constructs, have illuminated key mechanisms of injury, including axonal stretch, mitochondrial dysfunction, and neuroinflammation. However, limitations persist. PDMS-based devices remain prone to small-molecule absorption and limited long-term compatibility, while organoid models face challenges with vascularization, scalability, and reproducibility. The adoption of alternative biocompatible polymers such as cyclic olefin copolymers, thermo-plastic elastomers, and hybrid hydrogel–elastomer systems promises to enhance chemical stability and physiological realism.

Future progress will depend on advances in hydrogel, better cellular systems, mechanical stimulus, device design, imaging, and integration strategies. Combining these with emerging technologies such as 3D bioprinting, integrated biosensors, and vascularized organoids will create dynamic, human-relevant platforms for investigating injury progression and recovery modelling. By integrating AI-technologies microfluidic and tissue-engineered platforms, researchers can generate highly reproducible, physiologically relevant models, ultimately moving TBI research toward precision therapeutics and personalized injury mitigation strategies. Ultimately, the convergence of microfluidics, biomaterials, and tissue engineering holds the potential to move TBI research beyond modelling toward mechanism-driven, patient-specific therapeutic discovery. The NIH and FDA’s recent focus on developing and harmonizing new approach methodologies (NAMs) is transforming the landscape of drug testing and paving the way toward minimizing animal experimentation.

## Figures and Tables

**Table 1.** Summary of Microengineered and Multiorgan Chip Models Used for Traumatic Brain Injury Research.

Chip Model	Cells and Organs Used	Materials Used	Major Findings	Refs.
PDMS microfluidic device for TBI (Fabrication methods based on Huh, D., et al. (2010), Jin, H. J., et al. (2023))	Neural cells or 3D spheroids	PDMS (Shore A 40–50, 2–5 mm thickness), plasma-bonded to glass; soft-lithographed ECM-coated microchannels (collagen I, poly-L-lysine)	Recreates the physical forces of brain injury, showing tau build-up, TDP-43 changes	[38,39]
Microfluidic neurospheroid compression/stretch	iPSC-derived neurospheroids	PDMS membrane with hydrogel support (Matrigel and alginate)	Mimics mechanical strain seen in early TBI, leading to cell death and structural damage within neural networks.	[41,42]
Brain-on-chip (Dollé et al.)	Neurons with varying axon diameters	PDMS microchannels coated with ECM proteins	Shows that thinner axons are more vulnerable to injury, with mitochondrial stress as a key driver; NHE-1 inhibition helps protect cells.	[36]
Axons-on-a-chip (Pan et al.)	Neurons/isolated axons	PDMS microgrooves with poly-D-lysine and laminin	Models mild, repeated strain that causes localized axonal swellings and calcium imbalance without killing the cells.	[37]
Brain–lung multiorgan chip (Kim et al.)	Brain neurons + lung tissue	PDMS multichambered with porous PET membrane	Reveals how brain injury can trigger lung inflammation and how lung responses, in turn, worsen brain damage.	[43,44]
Brain–liver multiorgan chip (Materne et al.)	Human neurospheres + liver equivalents	PDMS-polycarbonate bioreactor with collagen ECM	Demonstrates that liver detoxification and metabolism influence how brain tissue recovers after injury.	[40]
Human cortical organoids-on-chip (Jgamadze et al.; Gautam & Agrawal)	3D brain organoids (neurons, astrocytes, microglia)	PDMS chip with hydrogel embedding (Matrigel and collagen I)	Captures brain-like layering and inflammation after injury; highlights the need for vascularized models.	[45]
Mechanical loading organoids (Beltrán et al.)	Human cortical organoids	PDMS stretchable platform with hydrogel matrix	Shows how mechanical strain triggers calcium spikes and activates genes linked to cell death and immune responses.	[46]
Blast-wave organoids (Bar-Kochba et al.)	Cortical organoids	PDMS chamber linked to pneumatic actuator; collagen and fibrin hydrogel	Simulates mild repetitive blast exposure, revealing subtle axonal damage and tau-related neuroinflammation.	[47]

**Table 2.** Elastic properties of brain tissue and representative hydrogel scaffolds.

Material/System	Elastic Modulus (Young’s)	Storage Modulus (G′)	Notes	References
Human Brain Tissue	~1 kPa	140–620 Pa	Region- and age-dependent	[68,69]
Neural Stem Cell Matrix	~0.1–1 kPa	Not reported	Promotes neuronal lineage	[70]
Glial Differentiation Zone	>1 kPa	Not reported	Stiffer, promotes astrocytes	[41]
Matrigel	~0.5–2 kPa	Not reported	Thermo-gelled ECM	[54]
GelMA	Tunable (0.5–20 kPa)	Not reported	Light-controlled	[42]
Alginate	Tunable (0.1–10 kPa)	Not reported	Ionic gelation	[36,58,66]
PEG-based Hydrogels	Highly tunable	Not reported	Synthetic, inert base	[37,58]

**Table 3.** In vitro and microphysiological models for studying traumatic brain injury (TBI).

Model Type/Example	Core Components (Neurons/Glia/Vasculature)	Injury Type Simulated	Advantages	Limitations	Applications
2D Stretch Model (hiPSC-derived neurons) [22]	Neurons (±astrocytes)	Uniaxial or biaxial stretch	Simple, high throughput; compatible with live imaging and molecular assays	Lacks 3D architecture; no neurovascular unit (NVU); limited chronic modeling	Primary injury mechanisms; neuroprotective drug screening
Microfluidic Neurospheroid Compression/Stretch [41,42,62]	iPSC-derived neurospheroids (neurons and glia)	Global compression or stretch via PDMS membrane or hydrogel	3D cell–cell interactions; physiologically relevant strain distribution	Limited vascularization; diffusion constraints	Early TBI cascades; network integrity; cell death and structural damage
Brain-on-a-Chip (Dollé et al.) [36,37]	Neurons with varying axon diameters	Controlled strain in microchannels	Reveals diameter-dependent vulnerability; insights into mitochondrial stress	Limited glial and vascular components; simplified extracellular matrix (ECM)	Axonal biomechanics; mitochondrial stress; pharmacological modulation (e.g., NHE-1 inhibition)
Axons-on-a-Chip (Pan et al.) [37]	Neurons with isolated axons	Localized shear or microfluidic jet-induced strain	Compartment-specific axonal injury; high-resolution imaging	Focused on axons only; lacks full tissue context	Diffuse axonal injury; focal swellings; calcium influx; cytoskeletal disruption
Human Cortical Organoids-on-Chip (Jgamadze et al.; Gautam & Agrawal) [45]	3D cortical organoids (neurons, astrocytes, microglia)	Compression, indentation, or mechanical insult	Recapitulates layered cortical architecture; captures inflammatory responses	Limited vascularization; batch-to-batch variability	Primary and secondary injury; gliosis; transcriptional responses
Mechanical Loading of Organoids (Beltran et al.) [46,47]	Human cortical organoids	Cyclic or static stretch on PDMS platforms	Tunable strain amplitude and frequency; links mechanics to gene expression	Requires calibration; limited vascular and immune components	Mechanogenomics; calcium dynamics; immune activation
Blast-Wave Organoids (Bar-Kochba et al.) [47]	Cortical organoids	Blast-like overpressure or shock wave	Models mild and repetitive TBI; captures subtle axonal and tau pathology	Complex setup; difficult to map strain fields	Mild/repetitive TBI; tau and TDP-43 pathology; long-term structural changes
Brain–Lung Multi-Organ Chip (Kim et al.) [43,44]	Brain neurons and lung tissue	Systemic inflammatory signaling via cytokine flow	Captures brain–lung crosstalk; models neurogenic pulmonary edema	High complexity; limited local brain mechanics	Systemic inflammation; multi-organ interactions; immune-mediated secondary injury
Brain–Liver Multi-Organ Chip (Materne et al.) [40]	Human neurospheres and liver equivalents	Secondary injury via metabolic and inflammatory factors	Integrates hepatic metabolism and detoxification	Requires specialized bioreactors; limited spatial resolution	Drug metabolism; neurotoxicity; recovery versus secondary injury
Vascularized Brain Organoids/Assembloids [15,31]	Brain organoids fused with vascular organoids (endothelial cells, pericytes, astrocytes)	Compression, stretch, or shear within vascularized 3D tissue	Incorporates NVU-like structures; supports perfusion and neuroinflammation	Technically demanding; variability in vascular integration	Blood–brain barrier-like function; neuroinflammation; chronic TBI; neurodegenerative progression

## Data Availability

Data are contained within the article.

## Abbreviations

The following abbreviations are used in this manuscript

2D: Two-Dimensional
3D: Three-Dimensional
AA: Acrylamide
AD: Alzheimer’s Disease
AI: Artificial Intelligence
Alg: Alginate
BBB: Blood–Brain Barrier
CD31: Cluster of Differentiation 31
CLARITY: Clear Lipid-exchanged Acrylamide-hybridized Rigid Imaging/Immunostaining-compatible Tissue Hydrogel
COC: Cyclic Olefin Copolymer
CTE: Chronic Traumatic Encephalopathy
CUBIC: Clear, Unobstructed Brain/Body Imaging Cocktails and Computational Analysis
DAI: Diffuse Axonal Injury
ECM: Extracellular Matrix
ExM: Expansion Microscopy
FPI: Fluid Percussion Injury
GelMA: Gelatin Methacrylate
G′: Storage Modulus
HA: Hyaluronic Acid
iPSC: Induced Pluripotent Stem Cell
iPSC-EC: iPSC-Derived Endothelial Cell
iPSC-MG: iPSC-Derived Microglia
iPSC-PC: iPSC-Derived Pericyte
LSFM: Light-Sheet Fluorescence Microscopy
MEA: Microelectrode Array
MEAs: Microelectrode Arrays
NAMs: New Approach Methodologies
NVU: Neurovascular Unit
PACT: Passive Clarity Technique
PDMS: Polydimethylsiloxane
PEG: Polyethylene Glycol
PET: Polyethylene Terephthalate
pTau: Phosphorylated Tau
SHIELD: Stabilization under Harsh Conditions via Intramolecular Epoxide Linkages to Prevent Degradation
TBI: Traumatic Brain Injury
TDP-43: TAR DNA-Binding Protein 43
UV: Ultraviolet
VEGF: Vascular Endothelial Growth Factor
VE-Cadherin: Vascular Endothelial Cadherin
vWF: von Willebrand Factor
